# Association between homocysteine level and unexplained recurrent pregnancy loss: a meta-analysis

**DOI:** 10.3389/fendo.2026.1855570

**Published:** 2026-06-18

**Authors:** Hui Du, Yuanbo Hu, Hong Ye, Song Di, Hongli Yan

**Affiliations:** Center of Reproductive Medicine, First Affiliated Hospital, Naval Medical University, Shanghai, China

**Keywords:** homocysteine, hyperhomocysteinemia, meta-analysis, risk factor, unexplained recurrent pregnancy loss

## Abstract

**Background:**

Unexplained recurrent pregnancy loss (uRPL) remains a major clinical challenge, with increasing evidence suggesting a potential role of hyperhomocysteinemia (HHcy) in its pathogenesis. However, findings from individual studies are inconsistent. A meta-analysis was therefore performed to summarize the findings.

**Methods:**

A systematic search of PubMed, Embase, and Web of Science was conducted to identify observational studies evaluating the association between elevated homocysteine (Hcy) levels and uRPL. Pooled odds ratios (ORs) with 95% confidence intervals (CIs) were synthesized using a random-effects model accounting for the potential influence of heterogeneity.

**Results:**

Thirteen case–control studies comprising 1,473 women with uRPL and 1,605 controls were included. Overall, HHcy was significantly associated with uRPL (OR 3.46, 95% CI 2.33–5.14; I² = 57%). Subgroup analyses showed consistent associations across geographic regions, with pooled ORs of 4.41 in Asia, 2.96 in Europe, and 2.59 in Africa/Middle East (*p* for subgroup difference = 0.58). The association was stronger in studies with Hcy cutoff > 12 μmol/L compared to ≤ 12 μmol/L (OR 4.02 vs. 2.00; *p* = 0.02), and in studies using HPLC or immunoassay compared with enzymatic methods (OR 3.59 and 4.53 vs. 1.84; *p* = 0.02). Meta-regression identified Hcy cutoff as a significant contributor to heterogeneity (coefficient = 0.044, *p* = 0.04; adjusted R² = 65.2%).

**Conclusions:**

Elevated Hcy levels are associated with increased odds of uRPL. Variations in Hcy cutoff definitions may partly explain between-study heterogeneity. These findings support HHcy as a potential risk indicator in uRPL, although further prospective studies are warranted.

**Systematic review registration:**

https://www.crd.york.ac.uk/PROSPERO/view/, identifier CRD420261368753.

## Introduction

Recurrent pregnancy loss (RPL) is a multifactorial reproductive disorder typically defined as two or more consecutive pregnancy losses before 20–24 weeks of gestation (1, 2). It affects approximately 2–5% of women of reproductive age and imposes substantial physical and psychological burdens on affected individuals and families (3). Despite extensive clinical evaluation, nearly 40–50% of cases remain unexplained after exclusion of established causes, including chromosomal abnormalities, uterine anomalies, endocrine disorders, and autoimmune conditions (4) This subgroup, commonly referred to as unexplained recurrent pregnancy loss (uRPL), highlights the critical need to identify additional and potentially modifiable risk factors (5, 6).

Homocysteine (Hcy) is a sulfur-containing amino acid involved in methionine metabolism and one-carbon pathways essential for DNA synthesis, methylation, and vascular function (7, 8). Under physiological conditions, Hcy levels are tightly regulated through remethylation and transsulfuration pathways, which depend on folate and B vitamins (9). Elevated Hcy levels, or hyperhomocysteinemia (HHcy), have been associated with endothelial dysfunction, oxidative stress, and a prothrombotic state (10, 11). In the context of pregnancy, these alterations may impair trophoblast invasion and placental vascularization, thereby contributing to early pregnancy failure (12, 13). Experimental evidence further suggests that HHcy may disrupt placental perfusion and chorionic villous development, providing a plausible biological mechanism linking Hcy to RPL (14, 15).

A growing body of observational studies has examined the relationship between Hcy and RPL (12). However, findings have been inconsistent. An early meta-analysis demonstrated that elevated Hcy was associated with an increased risk of recurrent early pregnancy loss, with pooled risk estimates ranging from 2.7 to 4.2 depending on measurement conditions (16). However, only 3 studies were included which affected the reliability of the finding (16). More recent meta-analyses have reported significantly higher Hcy levels in women with RPL compared with controls, although these analyses primarily used continuous outcomes and were limited by substantial heterogeneity and reduced clinical interpretability (17). Similarly, another meta-analysis confirmed that serum Hcy levels were elevated in patients with recurrent spontaneous abortion, but also highlighted limitations such as small sample sizes, heterogeneity in study design, and lack of standardized definitions (18). Importantly, prior meta-analyses did not focus specifically on uRPL, nor did they systematically evaluate clinically relevant categorical exposure (e.g., high vs low Hcy) or explore the impact of cutoff definitions on the observed associations (16–18). Given these limitations, an updated and methodologically refined meta-analysis is warranted. Therefore, the present study aimed to evaluate the association between elevated Hcy levels and uRPL using categorical exposure definitions, quantify the magnitude of this association, and explore potential sources of heterogeneity through subgroup and meta-regression analyses.

## Methods

The meta-analysis was carried out in accordance with established methodological guidance, following the principles outlined in the PRISMA 2020 statement (19) and the Cochrane Handbook for Systematic Reviews and Meta-Analyses (20), encompassing protocol planning, study selection, data collection, statistical analysis, and results interpretation. The study protocol was registered prospectively in the PROSPERO database (registration number: CRD420261368753).

### Database search

A systematic literature search was conducted in PubMed, Embase, and Web of Science to identify studies that met the eligibility criteria for inclusion. The search strategy was constructed using the combination of the following terms (1): “homocysteine” OR “hyperhomocysteinemia” OR “Hcy” OR “HHcy” OR “2-amino-4-mercaptobutyric acid”; and (2) “recurrent miscarriage” OR “recurrent abortion” OR “spontaneous abortion” OR “recurrent pregnancy loss” OR “recurrent fetal loss” OR “habitual abortion” OR “repeated miscarriage” OR “pregnancy wastage” OR “early pregnancy loss” OR “RPL”. Only full-text, peer-reviewed articles published in English and involving human participants were eligible for inclusion. Additionally, the reference lists of relevant reviews and original studies were manually examined to identify further potentially eligible publications. All databases were searched from their inception up to February 22, 2026. Detailed search strategies for each database are presented in **Supplementary File 1**.

### Study inclusion and exclusion criteria

The selection of studies was guided by the PICOS principle:

P (Population): The population of interest included women of reproductive age diagnosed with uRPL defined as two or more consecutive pregnancy losses after exclusion of known etiologies. Studies were required to clearly rule out identifiable causes such as chromosomal abnormalities, uterine structural defects, antiphospholipid syndrome, and endocrine disorders (e.g., polycystic ovary syndrome). Control groups consisted of women without a history of recurrent pregnancy loss or with at least one successful pregnancy.

I (Exposure): The exposure of interest was elevated Hcy levels (HHcy). Eligible studies defined exposure using a dichotomized classification (high vs. low Hcy) based on predefined or clinically relevant thresholds. Studies reporting only continuous Hcy levels without categorization were not considered for the primary analysis.

C (Comparator): The comparator group included participants with normal or lower Hcy levels within the same study population. This allowed for direct comparison between high and low exposure groups to estimate the association with uRPL. Studies were required to clearly define the reference (low Hcy) group.

Outcome (O): The primary outcome was the presence of uRPL. Studies had to report effect estimates as odds ratios (ORs) with 95% confidence intervals (CIs), or provide sufficient data (e.g., number of cases and controls with high vs low Hcy) to allow calculation of ORs. Only studies evaluating the association between Hcy and uRPL risk were included.

S (Study design): Eligible studies included observational designs, specifically case–control, cross-sectional, or cohort studies conducted in human populations. Only peer-reviewed original research articles were considered.

Studies were excluded if they failed to report dichotomized Hcy exposure (high vs low) or sufficient data to calculate ORs. Studies focusing exclusively on populations with known causes of pregnancy loss (e.g., PCOS, antiphospholipid syndrome, chromosomal abnormalities, or uterine anomalies) were excluded unless uRPL data could be clearly separated. Mechanistic, experimental, or preclinical studies (including animal models, cell experiments, or omics-based analyses) were excluded. Studies reporting Hcy only within RPL cases, without a control group, or only providing continuous data (e.g., mean ± SD) without dichotomization were also excluded. When multiple publications from the same population were identified, only the study with the most comprehensive data and the largest sample size was included to avoid duplication.

### Study quality assessment

Two reviewers independently performed the literature search, screened studies for eligibility, extracted data, and assessed study quality. Any disagreements were resolved through discussion, and when necessary, a third investigator was consulted to reach consensus. The methodological quality of the included studies was evaluated using the Newcastle–Ottawa Scale (NOS) (21), which assesses study quality across three domains: selection, comparability, and outcome assessment. NOS scores range from 1 to 9, with studies scoring ≥ 8 considered to be of high quality.

### Data collection

Data extraction was conducted independently by two reviewers using a standardized and pre-tested data collection form. Extracted variables included study characteristics (first author, publication year, country, and study design), participant characteristics (diagnosis of uRPL, definition of controls, numbers of cases and controls included, and mean ages of the included women), exposure details (timing and methods for measuring blood Hcy levels, methods for determining the cutoff values for defining HHcy, and cutoff values of HHcy), and variables matched or adjusted for in the analyses examining the association between HHcy and uRPL.

### Statistical analyses

The association between HHcy and uRPL was evaluated by pooling ORs with their corresponding 95% CIs (20), compared between women with high vs. low blood Hcy. If multiple models were reported, effect estimates from the most adequately adjusted model were preferentially extracted to minimize the influence of confounding. When necessary, effect estimates and their standard errors were derived from the reported 95% CIs or *p* values. Before pooling, all effect measures were converted to the natural logarithmic scale to improve normality and ensure variance stabilization for meta-analysis (20). To evaluate variability across studies, we applied the Cochrane Q test, and calculated the I² statistic (22) and the between-study variance (τ²) (20). Heterogeneity was categorized based on I² values as low (< 25%), moderate (25–75%), or high (> 75%). Pooled effect estimates were calculated using the inverse variance (IV) approach within a random-effects framework (DerSimonian–Laird method) to account for potential between-study variability (20). In addition, 95% prediction intervals (PIs) were calculated for the primary analysis to estimate the range of true effects expected in similar future studies, taking into account both within- and between-study variability (20). Sensitivity analyses were performed using a leave-one-out approach, in which each study was sequentially excluded to assess the stability and robustness of the pooled results (23). To identify possible sources of between-study variability, we performed predefined subgroup analyses stratified by study region, definition of uRPL (≥ 2 or 3 pregnancy losses), methods for measuring the blood Hcy level, methods for determining the cutoffs of Hcy, cutoff values for the diagnosis of HHcy in women, whether age was controlled, and study quality scores in NOS. A cutoff of 12 μmol/L was selected for subgroup analyses, as it approximates the upper limit of normal Hcy levels in women and is commonly used in the literature to distinguish elevated from normal concentrations (24). Additionally, univariate meta-regression analyses were conducted to examine the potential impact of study-level characteristics on the association between HHcy and uRPL in women, such as sample size, cutoffs of Hcy, and NOS scores (20). Publication bias was assessed through visual inspection of funnel plot symmetry and further evaluated using Egger’s regression test (25). A two-sided *p* value < 0.05 was considered statistically significant. All analyses were performed using RevMan (version 5.3; Cochrane Collaboration, Oxford, UK) and Stata (version 17.0; StataCorp, College Station, TX, USA).

## Results

### Database search results

**Figure 1:**
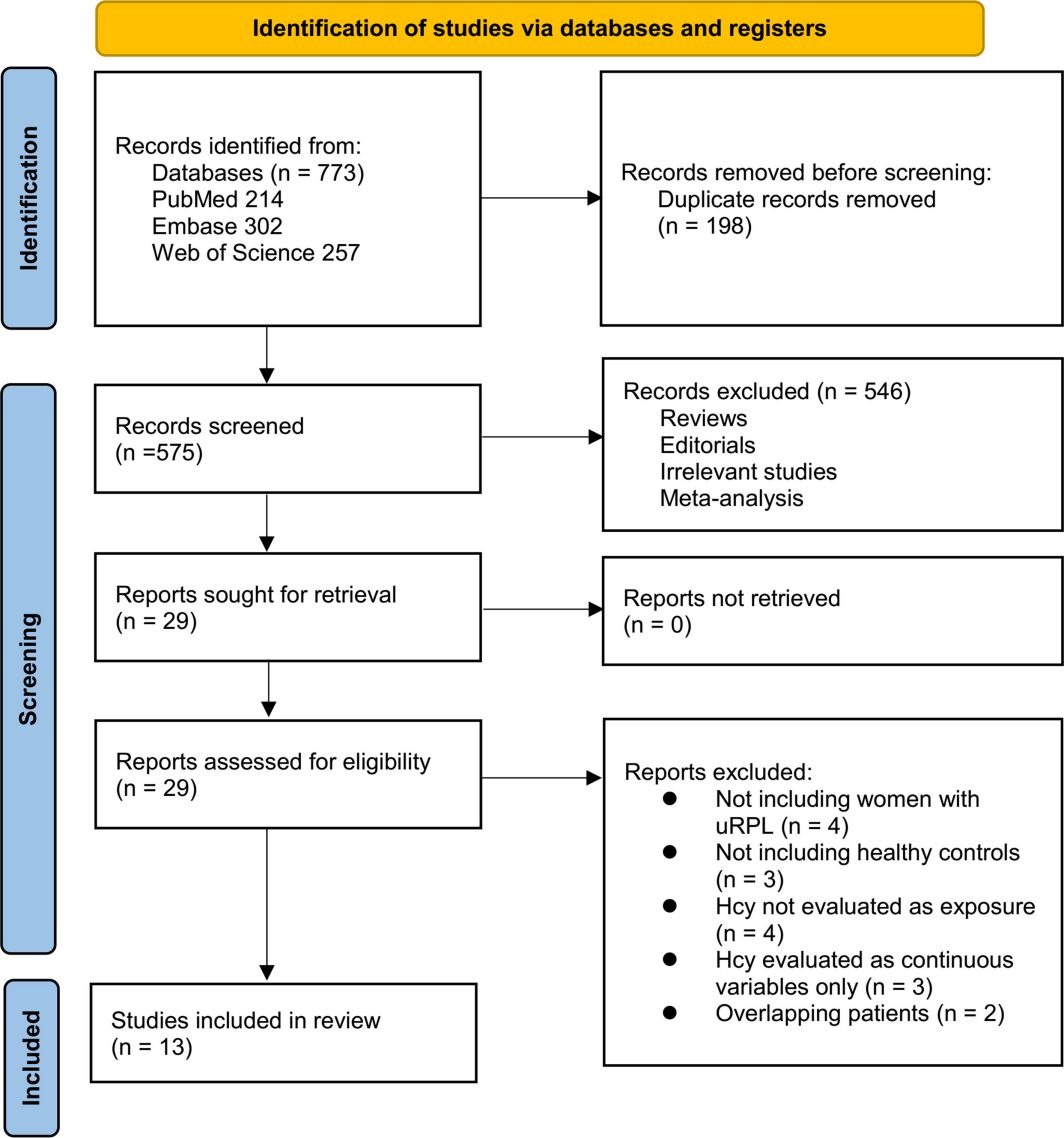
Flow diagram of the study selection process.

The study selection process is illustrated in **Figure 1**. A total of 773 records were retrieved from the three databases, of which 198 duplicates were removed. Following title and abstract screening, 546 records were excluded for not meeting the predefined inclusion criteria. The full texts of 29 articles were subsequently evaluated independently by two reviewers, and 16 were excluded for the reasons detailed in **Figure 1**. Ultimately, 13 studies met the eligibility criteria and were included in the quantitative meta-analysis (26–38).

### Overview of study characteristics

The main characteristics of the included studies are summarized in **Table 1**. A total of 13 case–control studies published between 1993 and 2025 were included in this meta-analysis, comprising 1,473 women with uRPL and 1,605 controls. All studies adopted a case-control design and defined uRPL as ≥ 2 (26, 28, 29, 34, 35, 37, 38) or ≥ 3 (27, 30–33, 36) consecutive pregnancy losses before 20-24 weeks of gestation, with exclusion of known etiologies. The included studies were conducted across diverse geographic regions, including Europe (The Netherlands, France), Asia (China, India, Pakistan, Saudi Arabia), Africa (Egypt, Tunisia), and the Middle East (Israel), reflecting broad population heterogeneity. The mean age of participants, where reported, ranged from approximately 25.2 to 34.7 years, indicating a predominantly reproductive-age population. In all studies, Hcy levels were measured under fasting conditions in the non-pregnant state at enrollment or after the most recent pregnancy, ensuring comparability across case and control groups. Various analytical methods were used for Hcy measurement, including high-performance liquid chromatography (HPLC) (26–30, 32), enzyme-linked immunosorbent assay (ELISA) (31, 35), chemiluminescence (33, 36, 37), and enzymatic assays (34, 38), which may contribute to inter-study variability. The definition of elevated Hcy varied across studies, with some using percentile-based cutoffs (e.g., 95th or 97.5th percentile of controls) (26–29, 32), while others adopted predefined thresholds from previous studies (30, 31, 33–37) or data-driven approaches (e.g., X-tile analysis) (38). The reported cutoff values ranged from 10.0 to 18.3 μmol/L, reflecting heterogeneity in exposure classification. Ten studies matched or adjusted for at least age (27, 29–33, 35–38), while several additionally controlled for variables including geographical factors, body mass index, lifestyle factors, and genetic polymorphisms (29–33, 38). Three studies did not match or adjust for age when the association between HHcy and uRPL was analyzed (26, 28, 34).

**Table 1:** Characteristics of the included studies.

Study	Country	Design	Diagnosis of uRPL	No. of women with uRPL	Definition of controls	No. of controls	Mean age (years)	Timing of Hcy measuring	Methods for Hcy measuring	Methods for defining the cutoff of Hcy	Cutoff of Hcy (μmol/L)	Variables matched or adjusted
Wouters 1993 (26)	The Netherlands	CC	≥2 consecutive spontaneous abortions within 16 weeks menstrual age; excluding known etiologies	102	Women with ≥1 live birth, no history of spontaneous abortion, fetal death, or placental abruption	41	NR (range: 22-47 years)	Non-pregnant, within 6 months after last pregnancy, at enrollment	HPLC	97.5th percentile of control group distribution	15	None
Quere 1998 (27)	France	CC	≥3 consecutive unexplained early pregnancy loss; excluding known etiologies	100	Matched healthy women with no antecedent fetal loss	100	NR	Non-pregnant, at enrollment	HPLC	95th percentile of control group distribution	10	Age
Coumans 1999 (28)	The Netherlands	CC	≥2 spontaneous abortions before 16 weeks gestation; excluding known etiologies	52	Healthy women with normal menstrual cycle and obstetric history of only uncomplicated pregnancies	67	NR	Non-pregnant, second half of normal menstrual cycle, at enrollment	HPLC	97.5th percentile in healthy premenopausal volunteers	16	None
Nelen 2000 (29)	The Netherlands	CC	≥2 spontaneous abortions before 16 weeks gestation; excluding known etiologies	123	Acquaintances of cases, comparable for age, geographical area, social class; had ≥1 liveborn infant and no spontaneous abortions	104	31.3	Non-pregnant, not lactating; median 3 months after last pregnancy, at enrollment	HPLC	95th percentile of control group distribution	18.3	Age and geographical area
Raziel 2001 (30)	Israel	CC	≥3 consecutive abortions; excluding known etiologies	36	Healthy non-pregnant women with 1–4 successful pregnancies, no thromboembolic history, matched for age and geographic origin	40	34.7	Non-pregnant, at enrollment	HPLC	Previous study defined	11	Age and geographical area
Mtiraoui 2006 (31)	Tunisia	CC	≥3 consecutive pregnancy loss at 5–30 weeks gestation; excluding known etiologies	200	Women matched for ethnic origin, no spontaneous pregnancy loss, uncomplicated pregnancy	200	28.5	Non-pregnant, at enrollment	ELISA	Previous study defined	15	Age, education, smoking, OC use, and BMI
Govindaiah 2009 (32)	India	CC	≥3 unexplained recurrent pregnancy losses (8–12 weeks or 8–20 weeks); excluding known etiologies	140	Women with normal reproductive history (≥2 children, no pregnancy loss)	140	NR (range: 18-35 years)	Non-pregnant, at enrollment	HPLC	95th percentile of control group distribution	14	Age and MTHFR genotype
Puri 2013 (33)	India	CC	≥3 consecutive unexplained pregnancy losses before 24 weeks; excluding known etiologies	83	Women with ≥2 consecutive normal deliveries, no pregnancy losses, matched for age, geography, social class, ethnicity	148	25.2	Non-pregnant, at enrollment	Chemiluminescence	Previous study defined	13	Age, geography, social class, ethnicity
Elagab 2022 (34)	Saudi Arabia	CC	≥2 failed pregnancies per ASRM definition; excluding known etiologies	58	Healthy women with at least two successful pregnancies and healthy babies, no history of pregnancy losses, pregnancy complications, chronic diseases, or vascular thrombosis	30	33.9	Non-pregnant, at enrollment	Enzymatic assay	Previous study defined	12	None
Zhu 2023 (36)	China	CC	≥3 consecutive unexplained pregnancy losses before 24 weeks; excluding known etiologies	50	Multiparous women with successful pregnancy, no abortion history, one birth	50	29.5	Non-pregnant, at enrollment	Chemiluminescence	Previous study defined	15	Age
Afaq 2023 (35)	Pakistan	CC	≥2 consecutive unexplained pregnancy losses before 20 weeks; excluding known etiologies	62	Age-matched women with ≥2 normal term deliveries, no history of abortion or other comorbidities	62	27.3	Non-pregnant, at enrollment	ELISA	Previous study defined	14	Age
Wang 2025 (38)	China	CC	≥2 consecutive pregnancy losses before 24 weeks; excluding known etiologies	272	Women with at least one normal delivery, no history of pregnancy loss	533	NR	Non-pregnant, at enrollment	Enzymatic assay	X-tile analysis determined	11.2	Age, TPO-Ab, TG-Ab, 25-(OH)D, and FA
Abd Elhameed 2025 (37)	Egypt	CC	≥2 failed clinical pregnancies (ASRM definition); excluding known etiologies	195	Healthy females <35 years, no pregnancy loss history, at least one uncomplicated full-term pregnancy	90	26.3	Non-pregnant, at enrollment	Chemiluminescence	Previous study defined	13.9	Age

NR, not reported; uRPL, unexplained recurrent pregnancy loss; CC, case–control; Hcy, homocysteine; HPLC, high-performance liquid chromatography; ELISA, enzyme-linked immunosorbent assay; BMI, body mass index; OC, oral contraceptive; MTHFR, methylenetetrahydrofolate reductase; ASRM, American Society for Reproductive Medicine; TPO-Ab, thyroid peroxidase antibody; TG-Ab, thyroglobulin antibody; 25-(OH)D, 25-hydroxyvitamin D; FA, folic acid.

### Study quality evaluation

The methodological quality of the included studies was assessed using the NOS score, with detailed results presented in **Table 2**. The NOS scores ranged from 6 to 9, indicating moderate to high overall study quality. Two studies achieved the maximum score of 9 (32, 38), reflecting strong methodological rigor, including adequate case definition, appropriate selection of controls, comprehensive adjustment for confounders, and reliable exposure assessment. Three studies scored 8 (29, 31, 33), reflecting generally robust selection and comparability, with minor limitations such as incomplete representativeness of cases or insufficient information on non-response rates. Six studies scored 7 (27, 28, 30, 35–37), primarily owing to insufficient control for confounding variables beyond age or limited representativeness of the study population. Two studies received a score of 6 (26, 34), mainly due to lack of adjustment for key confounders and potential selection bias. Overall, the included studies were considered to be of moderate to high methodological quality, supporting the validity of the pooled estimates, while acknowledging some heterogeneity in study design, exposure definition, and confounder adjustment.

**Table 2:** Study quality evaluation via the Newcastle-Ottawa Scale.

Study	Adequate definition of the cases	Representativeness of the cases	Selection of Controls	Definition of Controls	Controlled for age	Controlled for other factors	Ascertainment of exposure	Same method of ascertainment for cases and controls	Non-response rate	Overall
Wouters 1993 (26)	1	1	1	1	0	0	1	1	0	6
Quere 1998 (27)	1	1	1	1	1	0	1	1	0	7
Coumans 1999 (28)	1	1	1	1	0	0	1	1	1	7
Nelen 2000 (29)	1	1	1	1	1	1	1	1	0	8
Raziel 2001 (30)	1	0	1	1	1	1	1	1	0	7
Mtiraoui 2006 (31)	1	0	1	1	1	1	1	1	1	8
Govindaiah 2009 (32)	1	1	1	1	1	1	1	1	1	9
Puri 2013 (33)	1	1	1	1	1	1	1	1	0	8
Elagab 2022 (34)	1	0	1	1	0	0	1	1	1	6
Zhu 2023 (36)	1	1	1	1	1	0	1	1	0	7
Afaq 2023 (35)	1	1	1	1	1	0	1	1	0	7
Wang 2025 (38)	1	1	1	1	1	1	1	1	1	9
Abd Elhameed 2025 (37)	1	1	1	1	1	0	1	1	0	7

### Results of the meta-analysis

**Figure 2:**
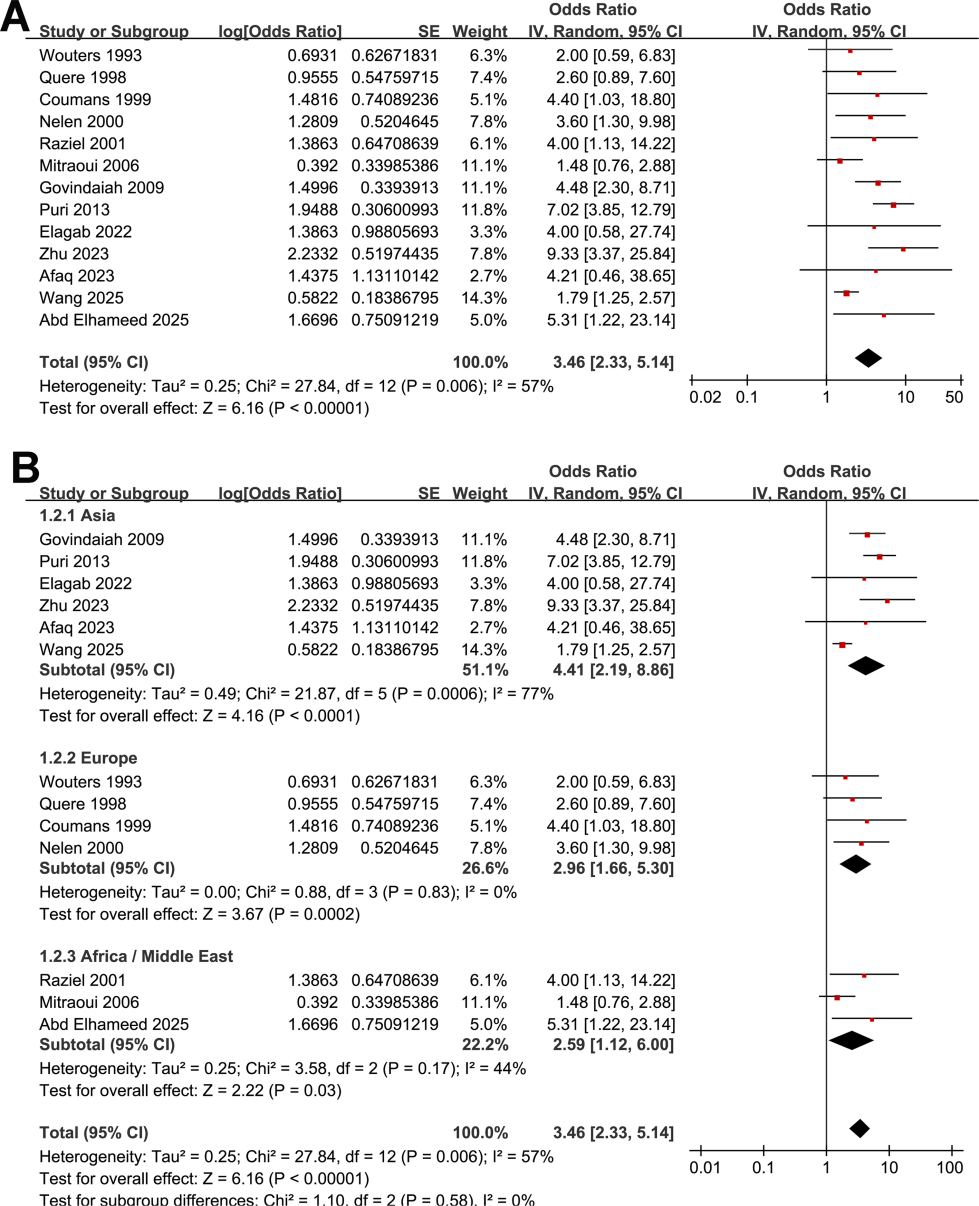
Forest plots showing the meta-analysis of the association between HHcy and uRPL: **(A)** forest plots for the overall meta-analysis; and **(B)** forest plots for the subgroup analysis according to study regions.

The pooled analysis of the included studies (26–38) demonstrated that HHcy was associated with uRPL in women (OR: 3.46, 95% CI: 2.33 to 5.14, *p* < 0.001; **Figure 2A**) with moderate heterogeneity (Cochrane Q test *p* = 0.006; I² = 57%; τ² = 0.25). The corresponding 95% PI ranged from 1.20 to 9.95, indicating that although the overall association was statistically significant, the magnitude of the effect may vary across different study settings. Leave-one-out sensitivity analyses yielded consistent results, with pooled ORs ranging from 3.03 to 3.87 (all *p* < 0.05), indicating the robustness of the overall estimate.

### Results of the subgroup analysis

**Figure 3:**
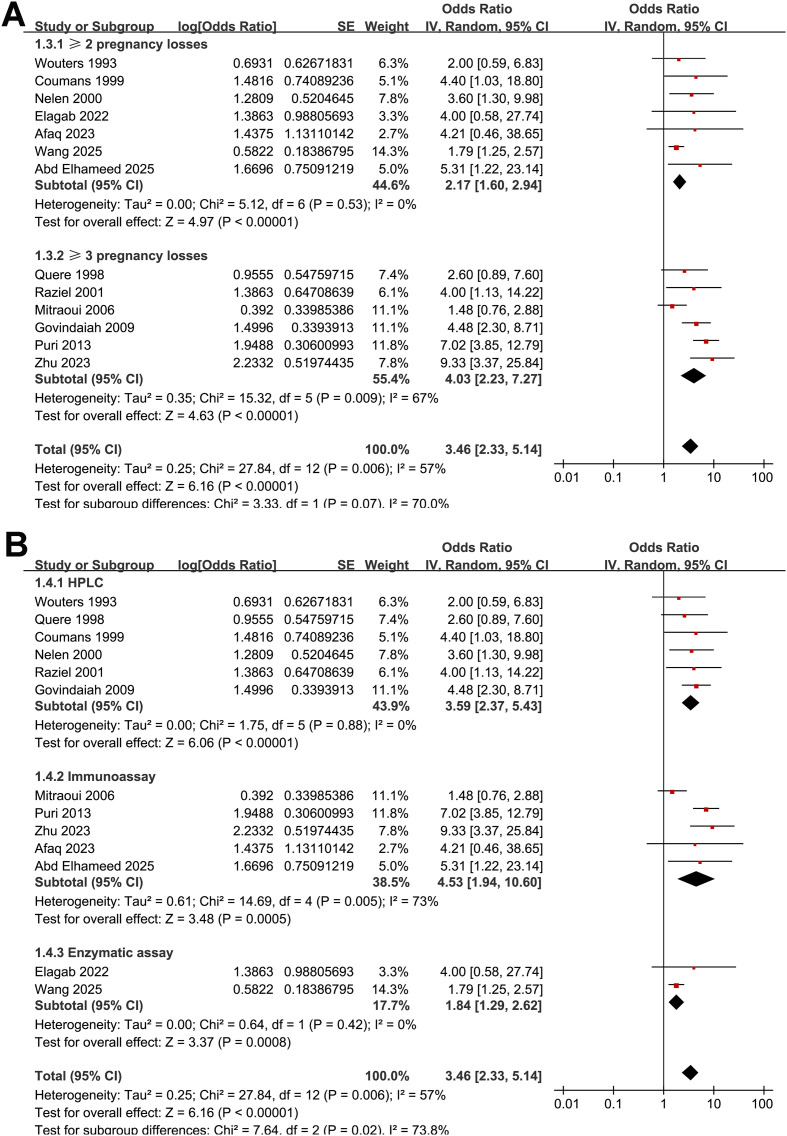
Forest plots showing the subgroup analysis of the association between HHcy and uRPL: **(A)** forest plots for the subgroup analysis according to the definition of uRPL; and **(B)** forest plots for the subgroup analysis according to methods for measuring Hcy level.

**Figure 4:**
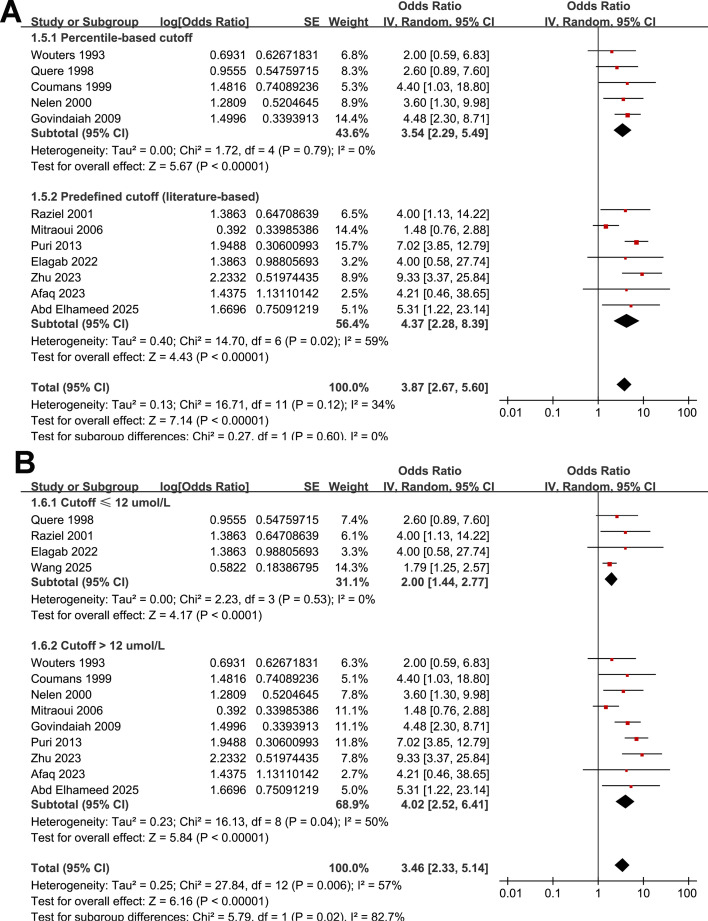
Forest plots showing the subgroup analysis of the association between HHcy and uRPL: **(A)** forest plots for the subgroup analysis according to the method for determining the cutoff of Hcy; and **(B)** forest plots for the subgroup analysis according to the cutoff values of Hcy.

**Figure 5:**
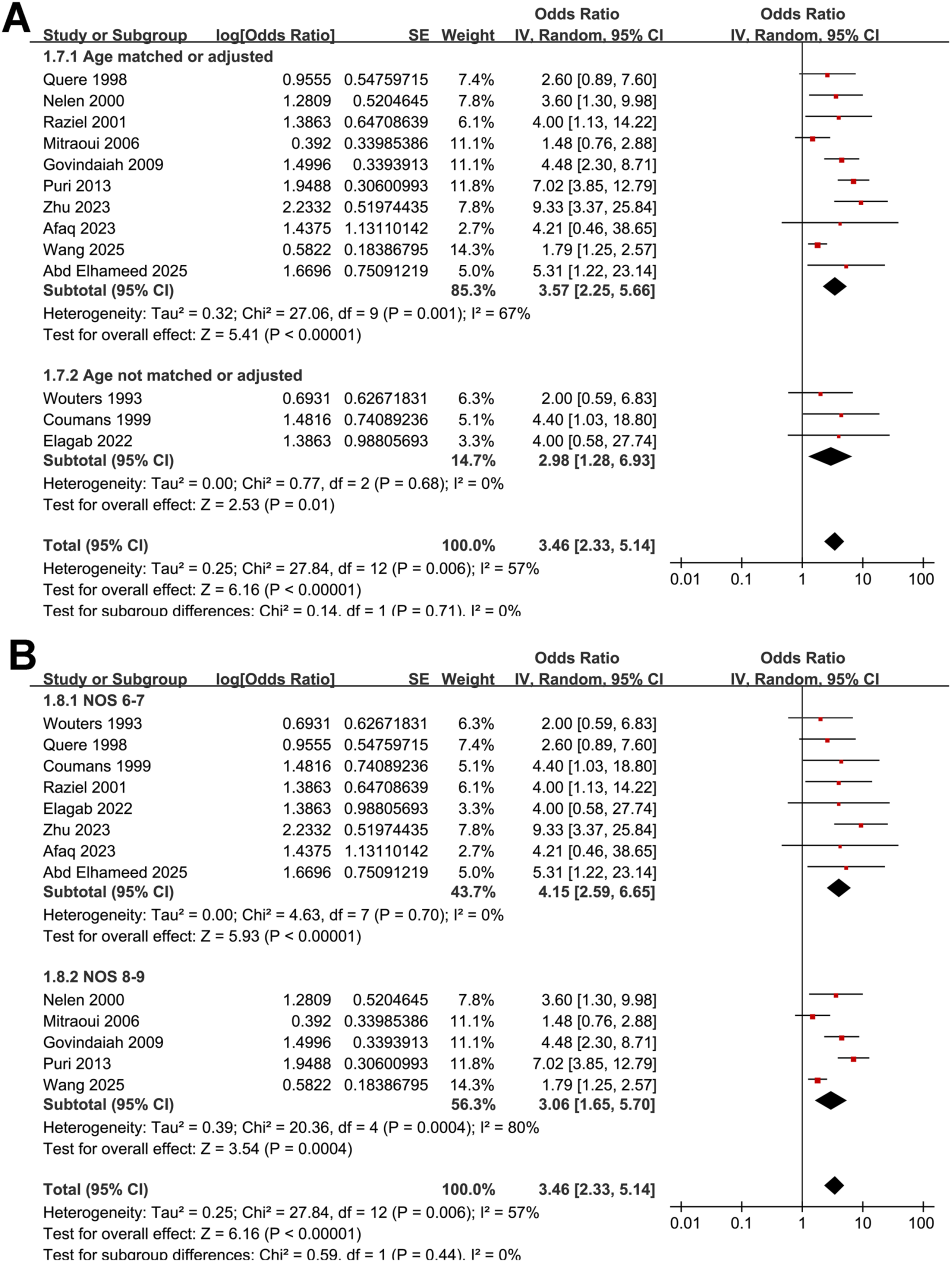
Forest plots showing the subgroup analysis of the association between HHcy and uRPL: **(A)** forest plots for the subgroup analysis according to whether age was matched or adjusted; and **(B)** forest plots for the subgroup analysis according to the study quality score.

Subgroup analyses suggested a similar association between HHcy and uRPL in women in studies performed in Asia, Europe, and Africa/Middle East (OR: 4.41, 2.96, and 2.59, respectively, *p* for subgroup difference = 0.58; **Figure 2B**). The results were not significantly different between studies with uRPL defined as ≥ 2 or ≥ 3 pregnancy losses (OR: 2.17 vs. 4.03, *p* for subgroup difference = 0.07; **Figure 3A**). A stronger association between HHcy and uRPL was observed in studies with blood Hcy measured with HPLC and immunoassay as compared to those with enzymatic assay (OR: 3.59, 4.53 vs. 1.84, *p* for subgroup difference = 0.02; **Figure 3B**). Similar results were observed for studies using percentile-based and literature-defined cutoffs of Hcy (OR: 3.54 vs. 4.37, *p* for subgroup difference = 0.60; **Figure 4A**). A stronger association between HHcy and uRPL was observed in studies with the cutoff of Hcy > 12 μmol/L compared to those ≤ 12 μmol/L (OR: 4.02 vs. 2.00, *p* for subgroup difference = 0.02; **Figure 4B**). Further analyses showed consistent results in studies with or without the control of age (OR: 3.57 vs. 2.98, *p* for subgroup difference = 0.71; **Figure 5A**), and in studies with the NOS of 6-7 and 8-9 (OR: 4.15 vs. 3.06, *p* for subgroup difference = 0.44; **Figure 5B**).

### Results of the meta-regression analysis

Univariate meta-regression analysis was performed to explore potential sources of heterogeneity, as summarized in **Table 3**. The results showed that the cutoff value of Hcy was positively associated with the effect estimate (coefficient = 0.044, *p* = 0.04), explaining a substantial proportion of between-study heterogeneity (adjusted R² = 65.2%). This finding suggests that variations in the definition of elevated Hcy may contribute importantly to the observed differences in effect sizes across studies. Sample size and NOS score of the included studies showed no significant association with the effect estimate (*p* = 0.24 and 0.57, respectively). Overall, these findings indicate that differences in Hcy cutoff values may be a key contributor to heterogeneity in the meta-analysis.

**Table 3:** Results of univariate meta-regression analysis.

Variables	OR for the association between Hcy and uRPL			
	Coefficient	95% CI	*p* values	Adjusted R^2^
Sample size	-0.0015	-0.0040 to 0.0010	0.24	0%
Cutoff of Hcy (μmol/L)	0.044	0.002 to 0.086	0.04	65.2%
NOS	-0.12	-0.58 to 0.34	0.57	0%

OR, odds ratio; CI, confidence interval; NOS, Newcastle-Ottawa Scale; Hcy, homocysteine; uRPL, unexplained recurrent pregnancy loss.

### Publication bias

**Figure 6:**
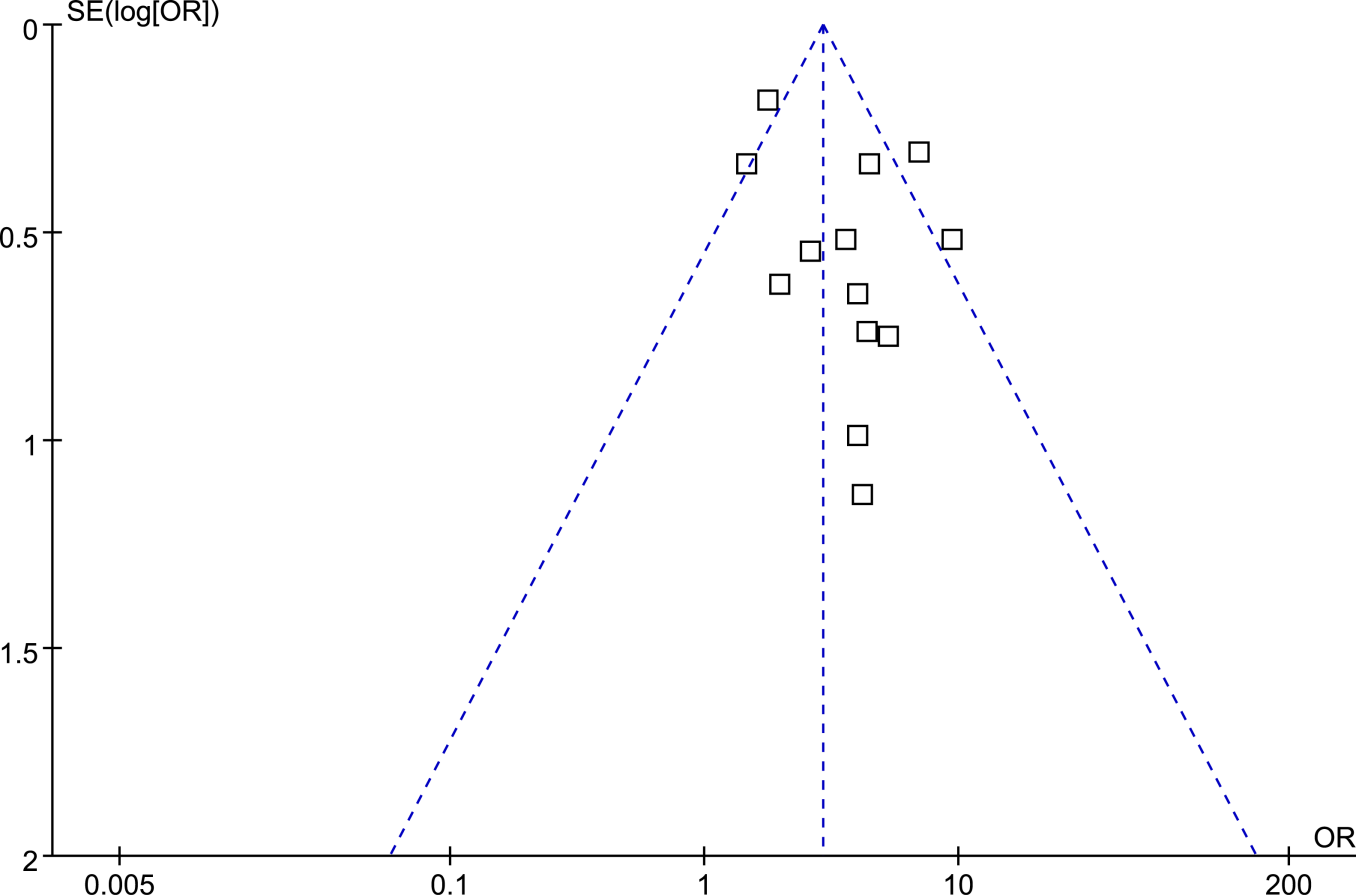
Funnel plots evaluating the publication bias for the meta-analysis of the association between HHcy and uRPL.

As illustrated in **Figure 6**, the funnel plots assessing the association between HHcy and uRPL in women appeared generally symmetrical. In line with this observation, Egger’s regression test did not detect significant publication bias (*p* = 0.65). Nevertheless, these findings should be interpreted with caution due to the relatively small number of included studies (k = 13).

## Discussion

The present meta-analysis provides comprehensive evidence supporting an association between elevated Hcy levels and the risk of uRPL. Rather than reiterating the quantitative findings, our results collectively indicate that Hcy may represent a biologically plausible and clinically relevant marker associated with impaired reproductive outcomes. This association appears consistent across most included studies, although its magnitude is influenced by methodological and clinical heterogeneity, particularly with respect to exposure definitions and measurement techniques.

Several biological mechanisms have been proposed that may help explain the observed association between HHcy and uRPL. First, vascular dysfunction appears to play a central role. Experimental and clinical evidence indicates that elevated Hcy exerts direct cytotoxic effects on endothelial cells, leading to apoptosis, increased membrane permeability, and the release of endothelial microparticles, thereby impairing vascular integrity (39, 40). These alterations may promote a prothrombotic state and compromise uteroplacental circulation, which is critical for early embryonic development (41). Supporting this, histopathological studies have demonstrated that elevated maternal Hcy levels are associated with significantly reduced chorionic villous vascular area, perimeter, and diameter, indicating defective placental vascularization (42). Such microvascular impairment may lead to insufficient oxygen and nutrient delivery, which is involved in embryonic demise.

Second, emerging evidence highlights the role of Hcy in placental development and cellular homeostasis (43). Preclinical studies have shown that HHcy is associated with impaired placental morphology and function, leading to reduced implantation rates and fetal viability, as well as impaired trophoblast proliferation and differentiation (14). These effects appear to be mediated through dysregulation of autophagy and lysosomal pathways, as well as altered gene expression profiles involved in placental growth (14). In addition, recent findings suggest that Hcy may induce ferroptosis in endometrial stromal cells via activation of the Mitogen-Activated Protein Kinase signaling pathway, thereby impairing endometrial receptivity and embryo implantation (44). These data collectively support a multifaceted pathogenic role of Hcy in early pregnancy, extending beyond vascular injury to include direct effects on placental and endometrial function.

Third, immunological mechanisms may also contribute to the association. Elevated Hcy levels have been linked to increased inflammatory responses and immune dysregulation (12). In women with RPL, HHcy has been associated with increased natural killer (NK) cell cytotoxicity, particularly in those with MTHFR gene polymorphisms (45). This enhanced immune activation may disrupt maternal–fetal tolerance, thereby increasing the risk of pregnancy failure (45). Furthermore, Hcy-induced inflammation and cytokine release may exacerbate endothelial dysfunction and thrombosis, reinforcing the interplay between immune and vascular pathways (46).

The subgroup and sensitivity analyses in this meta-analysis provide further insight into potential sources of heterogeneity. Notably, the association between Hcy and uRPL was more pronounced in studies using higher cutoff values (> 12 μmol/L) compared with those using lower thresholds (≤ 12 μmol/L), suggesting that more substantial elevations in Hcy may be required to exert clinically meaningful effects. This interpretation is further supported by the meta-regression analysis, in which the cutoff value was identified as a significant contributor to between-study heterogeneity. However, considerable variability existed in how HHcy was defined across studies, with cutoff values ranging from 10 to 18.3 μmol/L and derived either from population-based percentiles or predefined thresholds from prior literature. These differences may affect the comparability of results in several ways. First, percentile-based cutoffs are study-specific and depend on the distribution of Hcy levels in the control population, potentially leading to inconsistent exposure classification across studies. In contrast, predefined thresholds may improve between-study comparability but may not account for population-specific variations in Hcy levels. Second, heterogeneous cutoff definitions may introduce non-differential misclassification of exposure, which could attenuate or inflate the observed associations. Third, higher cutoff values may preferentially identify individuals with more pronounced metabolic or vascular disturbances, thereby contributing to larger effect sizes. Although subgroup and meta-regression analyses suggested that cutoff value was a significant contributor to heterogeneity, these findings should be interpreted cautiously, as residual variability in exposure definition may still influence the pooled estimates. Overall, the lack of standardized criteria for defining HHcy remains an important limitation and highlights the need for consensus-based thresholds in future studies to improve comparability and clinical interpretability.

In addition, differences in Hcy measurement methods appeared to influence the observed associations, with stronger effect estimates reported in studies using HPLC or immunoassay-based techniques compared with enzymatic assays, possibly reflecting differences in analytical sensitivity and exposure classification. Other subgroup analyses showed broadly consistent associations across geographic regions and definitions of uRPL (e.g., ≥ 2 vs ≥ 3 pregnancy losses), indicating that the observed relationship is relatively robust across different populations and diagnostic criteria. Similarly, sensitivity analyses demonstrated that the overall findings were not materially altered by the exclusion of individual studies, supporting the stability of the pooled estimates. In contrast, meta-regression analyses indicated that study-level characteristics such as sample size and methodological quality were not significantly associated with effect estimates, suggesting that these factors were less likely to account for the observed heterogeneity. Collectively, these findings highlight that variations in exposure definition and measurement, rather than differences in study design or population size, may be the primary drivers of heterogeneity in this meta-analysis.

From a clinical perspective, the findings of this meta-analysis suggest that Hcy may serve as a potentially modifiable biomarker associated with uRPL risk in women. Interventional studies have demonstrated that supplementation with folate, vitamin B6, and vitamin B12 can effectively reduce Hcy levels and may improve pregnancy outcomes in selected populations, particularly those with MTHFR mutations (47). While these findings are promising, they should be interpreted cautiously, as the available evidence is limited and not derived from large randomized controlled trials (RCTs). Nonetheless, assessment of Hcy levels may help identify high-risk individuals who could benefit from targeted nutritional or metabolic interventions.

This meta-analysis has several strengths. It incorporates an up-to-date and comprehensive literature search across multiple databases, includes a relatively large pooled sample size, and applies rigorous statistical methods, including subgroup and sensitivity analyses, to explore heterogeneity. Importantly, the use of categorical exposure definitions enhances the clinical interpretability of the findings compared with prior meta-analyses relying solely on continuous measures. However, several limitations should be acknowledged. First, all included studies were case–control in design, which are inherently susceptible to selection bias and cannot establish causality (48). Second, substantial heterogeneity was observed across studies, likely reflecting differences in demographic characteristics, dietary habits, lifestyle factors, and genetic backgrounds, such as MTHFR polymorphisms. These factors could not be fully accounted for due to the lack of individual participant data. Third, residual confounding remains an important concern. Many of the included studies did not comprehensively adjust for key factors known to influence Hcy levels, including folate and vitamin B12 status, BMI, smoking, renal function, and genetic determinants such as MTHFR polymorphisms (49). As a result, elevated Hcy may, at least in part, represent a surrogate marker of broader metabolic, nutritional, or inflammatory abnormalities rather than an independent causal factor for uRPL. This limitation is inherent to the observational design of the included studies and may bias the estimated association despite the use of adjusted models in some analyses. Therefore, the findings should be interpreted cautiously, and future studies with more comprehensive control of confounding variables are warranted to clarify the independent role of Hcy. Importantly, given the observational case–control design of the included studies, the present findings should be interpreted as evidence of association rather than causation. Finally, publication bias cannot be entirely excluded, although sensitivity analyses suggested that the overall findings were relatively robust.

Given these limitations, the results of this meta-analysis should be interpreted as evidence of association rather than causation. Future research should focus on well-designed prospective cohort studies and RCTs to clarify the causal role of Hcy in uRPL and to evaluate whether lowering Hcy levels can improve reproductive outcomes. Standardization of Hcy measurement methods and cutoff values is also needed to enhance comparability across studies. In addition, mechanistic studies integrating vascular, immunological, and metabolic pathways may further elucidate the complex role of Hcy in early pregnancy.

## Conclusions

In conclusion, the present meta-analysis suggests that elevated Hcy levels are associated with an increased risk of uRPL. While the underlying mechanisms likely involve a combination of vascular dysfunction, placental impairment, and immune dysregulation, the observational nature of the evidence precludes causal inference, and the findings should be interpreted as associative rather than causal. Hcy may represent a useful biomarker for risk stratification, but further research is required to determine its role in guiding clinical management and therapeutic interventions.

## Data Availability

The original contributions presented in the study are included in the article/**Supplementary Material**. Further inquiries can be directed to the corresponding author.
