# Supplementary material for: Association between homocysteine level and unexplained recurrent pregnancy loss: a meta-analysis
Source: Front Endocrinol (Lausanne). 2026 Jun 18;17:1855570. doi: 10.3389/fendo.2026.1855570 (PMC13322936; doi:10.3389/fendo.2026.1855570)
Supplement: Supplementary file 1 [file DataSheet1.docx]

**Detailed search strategy for each database**

**PubMed**

#1 "Homocysteine"[Mesh] OR homocysteine[tiab] OR hyperhomocysteinemia[tiab] OR Hcy[tiab] OR HHcy[tiab] OR "2-amino-4-mercaptobutyric acid"[tiab]

#2 "Abortion, Habitual"[Mesh] OR "Abortion, Spontaneous"[Mesh] OR "Pregnancy Complications"[Mesh] OR recurrent miscarriage[tiab] OR recurrent abortion[tiab] OR spontaneous abortion[tiab] OR recurrent pregnancy loss[tiab] OR recurrent fetal loss[tiab] OR habitual abortion[tiab] OR repeated miscarriage[tiab] OR pregnancy wastage[tiab] OR early pregnancy loss[tiab] OR RPL[tiab]

#3 #1 AND #2

**Embase**

#1 'homocysteine'/exp OR homocysteine:ti,ab OR hyperhomocysteinemia:ti,ab OR hcy:ti,ab OR hhcy:ti,ab OR '2 amino 4 mercaptobutyric acid':ti,ab

#2 'recurrent abortion'/exp OR 'spontaneous abortion'/exp OR 'pregnancy loss'/exp OR recurrent miscarriage:ti,ab OR recurrent abortion:ti,ab OR spontaneous abortion:ti,ab OR recurrent pregnancy loss:ti,ab OR recurrent fetal loss:ti,ab OR habitual abortion:ti,ab OR repeated miscarriage:ti,ab OR pregnancy wastage:ti,ab OR early pregnancy loss:ti,ab OR rpl:ti,ab

#3 #1 AND #2

**Web of Science**

TS = ((homocysteine OR hyperhomocysteinemia OR Hcy OR HHcy OR "2-amino-4-mercaptobutyric acid") AND ("recurrent miscarriage" OR "recurrent abortion" OR "spontaneous abortion" OR "recurrent pregnancy loss" OR "recurrent fetal loss" OR "habitual abortion" OR "repeated miscarriage" OR "pregnancy wastage" OR "early pregnancy loss" OR RPL))
